# Functional Chitosan Derivative and Chitin as Decolorization Materials for Methylene Blue and Methyl Orange from Aqueous Solution

**DOI:** 10.3390/ma12030361

**Published:** 2019-01-24

**Authors:** Abdelkader Labidi, Asier M. Salaberria, Susana C. M. Fernandes, Jalel Labidi, Manef Abderrabba

**Affiliations:** 1 Preparatory Institute of Scientific and Technical Studies of Tunis, University of Carthage, Sidi Bou Said road, B.P. 51 2070, La Marsa, Tunisia; abdelkaderlabidi0907@gmail.com (A.L.); abderrabbamanef@gmail.com (M.A.); 2 Chemistry Department, University of Sciences of Tunis, El Manar University, B.P: 248, El Manar II, 2092, Tunis, Tunisia; 3 Department of Chemical and Environmental Engineering, University of the Basque Country (UPV/EHU), Plza. Europa1, 20018 Donostia-San Sebastian, Spain; asier.martinez@ehu.eus; 4 CNRS/ Univ Pau & Pays Adour, Institut des Sciences Analytiques et de Physico-Chimie pour l’Environnement et les Materiaux, Umr 5254, 64000 Pau, France; susana.fernandes@univ-pau.fr

**Keywords:** Chitin, chitosan, polyacrylamide, dyes, decolorization, desorption

## Abstract

Dyes are classified as one of the major pollutants of water. They have negative impacts not only on environment but also on human health. In fact, wastewater that contains these harmful substances requires many types of treatments. Therefore, alternative methods and adsorption agents are needed. Herein, we propose to evaluate the decolorization of methylene blue (MB) and methyl orange (MO) as two models of soluble dyes from water using chitin and chitosan-graft-polyacrylamide. Furthermore, the applicability of these biomacromolecules as alternative adsorption agents, their sticking probability and desorption were also examined. Experimental parameters such as dye concentration, contact time, pH solution, adsorbent dosage and temperature were thoroughly examined for the grafted chitosan and chitin. The activation energy ($E_{a}$) and the thermodynamic variables (i.e., standard Gibb’s free energy ($\Delta G^{0}$), standard enthalpy ($\Delta H^{0}$), and standard entropy ($\Delta S^{0}$)) were determined using the Van’t Hoff and Arrhenius equations. The sticking probability (S*) model for MB and MO removal by chitin and the chitosan derivative demonstrated that both dyes were successfully removed under the proposed conditions. Desorption studies of MB and MO showed the reusability of both materials, suggesting their application for removing dyes from aqueous solution.

## 1. Introduction

**Figure 1:**
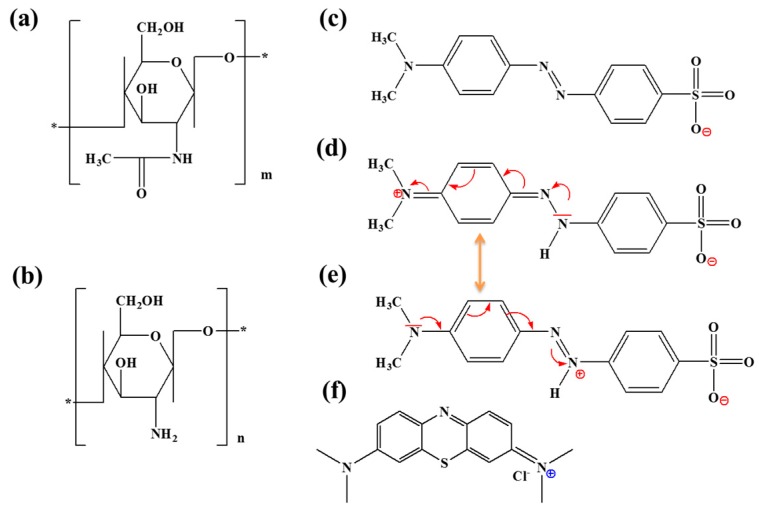
Chemical structure of: chitin (**a**); chitosan (**b**); anionic (**c**); zwitterionic forms of MO (**d**,**e**); and zwitterionic form of MB (**f**).

Water and wastewater can be contaminated by different pollutants due to the presence of many emerging compounds such as Ibuprofen (IBP) [1], heavy metals, volatile organic compounds (VOC) [2], and contaminants of emerging concern (CECs) [3]. The water-soluble dye methylene blue (C_16_H_18_N_3_SCl, Figure 1f) is commonly used in various fields such as dye manufacturing industries, plastics, cosmetics, rubber and printing [4]. MB is toxic, recalcitrant and carcinogenic, causing many health problems such as shock, vomiting, jaundice, etc. [5]. In addition, azo dyes have been considered as the largest family of dyes and colorants in textile application. They are also used to color food and beverages, candy and cosmetics. Among the azo dyes, methyl orange (C_14_H_14_N_3_NaO_3_S, Figure 1c–e), a water-soluble compound containing an azo group (–N=N–) is widely used in chemistry, dyeing, printing textiles and paper industries. This soluble dye can cause hypersensitivity, allergies and even endanger human health [6]. Consequently, the release of these dyes (MB and MO) in wastewater is a serious environmental problem. Several techniques have been used for their elimination such as photodecomposition, membrane filtration, electrochemical oxidation, biological treatment and adsorption [7,8,9,10,11].

Chitin is found in the shells of crustaceans. Containing the (1,4)-β-N-acetyl-D-glucosamine-repeating units makes it a marine product with interesting flocculating effect in water purification.

Chitin and its main derivative, chitosan, have been used in different applications in biomedicine, pharmacy, agriculture, textiles, wastewater treatment, environmental protection, antifungal treatment, etc. [12,13,14]. As is or modified, both biopolymers have been widely studied for the removal of pollutants [15,16,17,18]. Several chitosan derivatives have been designed using epichlorohydrin [19], glutaraldehyde [20], zeolites, etc. [21,22,23]. Based on previous research reports, the grafting of vinyl monomers on chitosan-based material has been investigated (i.e., poly vinyl chloride, poly vinyl alcohol, and poly(methacrylic acid)) [24,25,26]. Nonetheless, few studies on the adsorption of organic pollutants, in particular MB and MO, on chitin and grafted chitosan have been developed. Recently, chitosan ia used as adsorbent material of an anionic dye “Reactive Black 5” [27], and chemical cross-linked chitosan beads, chitosan-graft poly(methyl methacrylate) and chitosan magnetic composite are used for removal of azo dyes [28,29,30].

The objective of this work was to prepare a low cost and eco-friendly bioadsorbent (chitosan-graft-polyacrylamide). To the best of our knowledge, this chitosan derivative was used for the first time for methylene blue and methyl orange removal. The grafted chitosan was chosen due to the presence of good chelating groups (hydroxyl, amide and amino groups). In addition, chitin was extracted in our laboratory and used as renewable material to remove both dyes. The decolorization efficiency of methylene blue and methyl orange by chitin was investigated for comparative purpose under the same conditions. 

Experimental conditions, namely pH, concentration of MB and MO in water solution, contact time, temperature and various adsorbent doses, were examined. The thermodynamic and kinetic parameters of MB and MO decolorization by chitin and chitosan-grafted-polyacrylamide were also studied in detail. Furthermore, to assess the applicability of these biomacromolecules as alternative adsorption agents, their sticking probability was also examined and adsorption–desorption cycles were performed.

## 2. Experimental

### 2.1. Biopolymers and Chemical Compounds

Chitin (CH) powder was extracted in our laboratory from yellow lobster wastes (kindly supplied by Antartic Seafood S.A., Chile, Figure 1a). The degree of deacetylation was found to be 4% by ^13^C NMR. Chitosan powder (CS, degree of deacetylation of 98% and viscosity-average molecular weight of 500,000, Figure 1b) was supplied by Mahtani Chitosan PVT. Ltd., India and it was purified before use by a precipitation method [31], Sodium hydroxide (NaOH, reagent grade, ≥98%, pellets (anhydrous)), acetic acid (Ph. Eur. reagent ≥99.5%), nitric acid (HNO_3_, ACS reagent, 37%), hydrochloric acid (HCl, ACS reagent, 37%), acrylamide (Acros Organics, >99%), methanol (Ph. Eur. reagent >99%), cerium ammonium nitrate (CAN, Sigma Aldrich >99%), cationic dye (methylene blue) (MW 319.85 g/mol, anhydrous basis, >97%, Figure 1f) and Anionic dye (methyl orange) (MW 327.33 g/mol, ACS reagent 85%, Figure 1c,d) were supplied by Sigma-Aldrich and used as received without further purification. 

### 2.2. Synthesis of Chitosan-Grafted-Polyacrylamide

**Figure 2:**
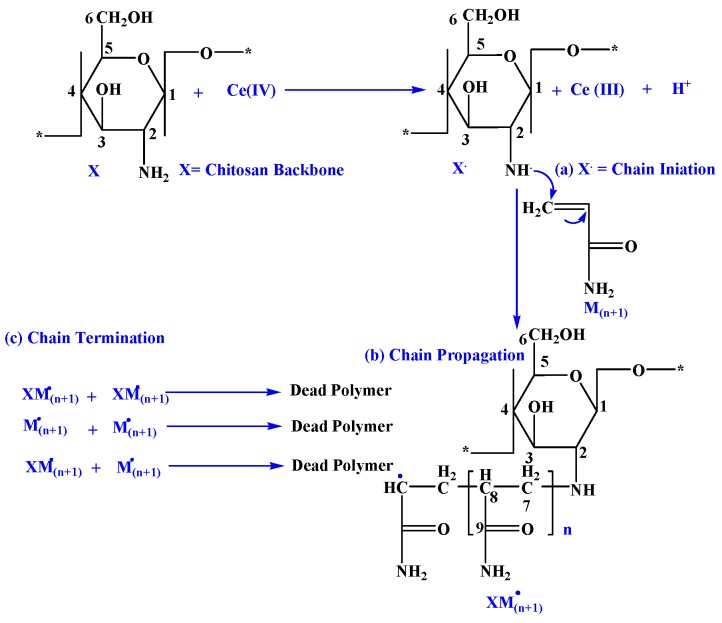
Mechanism of the graft copolymerization of acrylamide on chitosan backbone, where X represents chitosan and M represents acrylamide monomer.

The synthesis of chitosan-grafted-polyacrylamide was done as reported previously [32,33,34]. Briefly, first a determinate weight of chitosan (0.5 g) was stirred in 50 mL of 1% acetic acid solution. After dissolution, Ce (IV) (0.2 g in HNO_3_ (10 mL; 0.2 M) was added drop-wise over a period of 10 min under N_2_ atmosphere for the initiation of the free radical reaction. Then, acrylamide (1 g in 10 mL of distilled water) was added drop wise for 10 min at 50 °C for the copolymerization. Afterwards, the solution was stirred for 4 h. Then, the polymerization was stopped and the final sample was precipitated in acetone and purified with methanol to remove residual agents and a gel-like sample was obtained. The gel was then neutralized to pH 6.5–7.0 using 0.1 M NaOH and dried at 50 °C for 48 h in a vacuum oven (Figure 2). The calculated percentage of grafting (%G) was determined as:

(1) $$\%G=\frac{\mathrm{final}\;\mathrm{chitosan}\;\mathrm{weight}-\mathrm{intial}\;\mathrm{chitosan}\;\mathrm{weight}}{\mathrm{intial}\;\mathrm{chitosan}\;\mathrm{weight}}*100$$

### 2.3. Characterization of the Synthesized Chitosan Graft Polyacrylamide 

ATR-FTIR spectra were recorded on a Nicolet Nexus 670 equipped (Waltham, MA, USA) with a KRS^−5^ crystal of refractive index 2.4 and using an incidence angle of 45°. The spectra were taken in a transmittance mode in the wavenumber range of 750–4000 cm^−1^, with resolution of 4 cm^−1^ and after 128 scan accumulations. 

^13^C solid-state NMR spectra were obtained using a Bruker 400 WB Plus spectrometer (Billerica, MA, USA). Spectra were collected by using a 4 mm CP-MAS probe with a sample spinning rate of 10 000 Hz. ^13^C CP-MAS spectra at 100.6 MHz of the solid samples were obtained using 12 h spectral accumulation time, a time domain of 2 K points, a spectral width of 29 kHz, a contact time of 1.5 ms and an interpulse delay of 5 s.

Thermogravimetric analysis (TGA) was carried out using a TGA/SDTA 851 Mettler Toledo instrument (Schwerzenbach, Switzerland). The scanning rate was about of 10 °C/min, from room temperature to 900 °C under nitrogen atmosphere (20 mL/min) using around 5 mg of each sample. Thermal property of the samples was detected by Differential Scanning Calorimeter (DSC, Mettler Toledo 882 e/700) (Schwerzenbach, Switzerland) using a constant heating rate of 5 °C/min for the temperature rise from −50 °C to 200 °C. Aluminum crucibles with pins and lids were employed as sample containers.

X-ray diffraction patterns were measured with a Philips X’pert Pro automatic diffractometer (Almelo, The Netherlands) using Cu-Ka radiation (operating at 40 kV and 40 mA) over the angular range of −70°.

Scanning electron microscopy images were obtained with a Scanning electron microscope JEOL JSM-6400F (Akishima, Tokio, Japan) with field emission cathode, a lateral resolution of 10–11 Å at 20 kV.

### 2.4. Decolorization Procedure

#### 2.4.1. Decolorization Experiments and Measurement of Dyes Concentration

One gram per liter MB and MO stock solutions were prepared by dissolving 0.1 g of each solid dye (MB and MO) in 100 mL of distilled water. Different concentrations ranging from 1.0 to 10.0 mg/L were prepared by dilution of the stock solution. The decolorization of MB and MO was studied in a concentration range of 1.0–10 mg/L (pH 8.0 and 5.0 for MB and MO, respectively, optimal pH for the decolorization) using 0.3 g·100 mL^−1^ of chitin and Chitosan-grafted-polyacrylamide (CS-g-PAM). MB and MO concentration was measured by colorimetric method using UV-visible spectrophotometer (Aquarious CECIL CE 7400) (Cambridge, UK) at 664 and 464 nm for MB and MO, respectively, corresponding to the maximum absorbance of both dyes [35,36].

The decolorization efficiency (DE) of MB and MO was defined as follows [37]:

(2) $$\mathrm{DE}=\left(1-\frac{C_{t}}{C_{0}}\right)*100,$$

$C_{0}$ and $C_{t}$ (mg/L) denote the initial concentration of dyes (MB and MO, respectively) and at each time (*t*).

#### 2.4.2. Evaluation of the Initial pH Effect, Adsorbents Concentration, Temperature and Regeneration Tests on Dyes Decolorization by Chitin and CS-g-PAM

The initial dyes concentration was 8 mg/L and chitin and CS-g-PAM was 3 g/L. The solution pH was varied in the range 2–10, and was adjusted by adding 0.1 M HNO_3_ and 0.1 M NaOH.

The effect of the used adsorbents (chitin and CS-g-PAM) concentration was assessed using a concentration range from 0.1 to 0.7 g·100 mL^−1^. This study was performed using an initial dyes concentration of 5 mg/L, the MO pH was 5.0 and the MB pH was 8.0 at 25 °C for 30 min. 

To find the endothermic or exothermic nature of the decolorization of MB and MO in aqueous solution, temperature was varied between 25 and 55 °C for an initial dye concentration of 4 mg/L at pH 8.0 and 5.0 for MB and MO, respectively.

The blue color of the two materials caused by the decoloraization of MB water solution was removed by its desorption using HCl (50 mL: 0.1 M) as eluent [38]; the desorption studies were performed by immersing the chitin and CS-g-PAM after MB adsorption in the acidic solution for a period of 2 h. Washed CH and CS-g-PAM powders were then used again for MO removal. These experiments were repeated for three cycles. 

The regeneration study of CH and CS-g-PAM after MO decolorization was performed using NaOH, as described in previous work [38]. Both chitin and CS-g-PAM powders, after MO removal, were stirred for 2 h in NaOH solution (50 mL of 0.1 M) for desorption of MO leading to obtaining a chitin and CS-g-PAM unloaded MO. Then, the obtained powder after desorption were washed with distilled water and used again for MO removal for three successive cycles. 

## 3. Results and Discussion

### 3.1. Characterization of the Chitosan Derivative (CS-g-PAM): Chemical Structure and Thermostability

**Figure 3:**
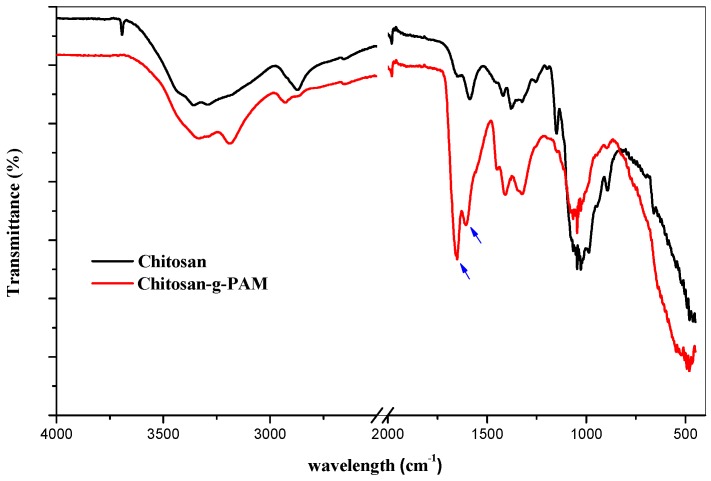
ATR-FTIR spectra of chitosan and chitosan-g-polyacrylamide (CS-g-PAM).

ATR-FTIR and ^13^C NMR spectra were used to confirm the successful grafting of the polyacrylamide onto chitosan chains. ATR-FTIR spectrum of chitosan (Figure 3) displays characteristic bands at 1557 cm^−1^, related to NH bending (amide II); at 1650 cm^−1^, which represented the carbonyl stretching (amide I) due to partial deacetylation; and intense bands at 1005–1060 cm^−1^, which were which mainly represented the CO stretching of the COH, CH_2_OH and COC groups in the chitosan backbone. The band at 3434 cm^−1^ could be related to the stretching vibration of O–H, N–H and inter hydrogen bonds of chitosan. The ATR-FTIR spectrum of the chitosan derivative (CS-g-PAM, %G = 300 %) shows characteristic peaks at 1665 and 1550 cm^−1^ related to the amide I and II bands, respectively, and a new peak at 1430 cm^−1^, which represented the C-N stretching in the obtained grafted chitosan [33,34].

Figure S1 shows the solid-state NMR, according to the literature [39]. Chitosan showed the flowing peaks: δ = 25 ppm attributed to the carbon (methyl) of the acetamido groups (chitosan not completely deacetylated); δ = 58 ppm attributed to the C_6_ and C_2_ carbons; δ = 75 ppm attributed to the C_5_ and C_3_ carbons; δ = 81 ppm due to the C_4_ carbon; δ = 102 ppm corresponding to the C_1_ carbon; and finally δ = 180 ppm due the C=O of the acetamido groups (chitosan not completely deacetylated) (according to the numeration of chitosan structure in Figure 2). In the spectra of the chitosan derivative (CS-g-PAM, Figure S1), the peak at δ = 41 ppm related to CHCH_2_ (C_7_) groups formed during the grafting of acrylamide on chitosan. The presence of a very intense peak at δ = 179 ppm corresponded to the carbon atoms of CONH_2_ (C_9_) group [40,41].

Figures S2 and S3 depict the thermal analyses (TGA and DSC) of chitosan and chitosan-g-PMMA to study the thermal proprieties of chitosan before and after grafting. Chitosan thermogram exhibit its typical thermal degradation profile with an initial temperature of degradation of 233 °C and a maximum decomposition rate around 264 °C. Grafted chitosan thermograms show four-stage decomposition with different mass rate loss compared to the unmodified chitosan. DSC (Figure S3) was measured to determine the phase behavior and thermal transitions of CS and CS-g-PAM materials. This revealed that the endothermic peak of chitosan at 62.84 °C increased for 55.25 °C after chemical modification. These results confirm the success of the reaction.

To study the morphology and crystallinity of the adsorbents, the powders were analyzed by SEM and X-ray diffraction. Figure S4a,b illustrates the SEM analysis of chitin and chitosan. As can be seen, chitin has a smooth surface; however, chitosan exhibits a rough surface. The SEM micrograph of chitosan-grafted-polyacrylamide obtained by Saha et al. [42] indicates that the surface of CS-g-PAM is rougher than chitosan. This observation can be attributed to the grafting of polyacrylamide on chitosan backbone. These results were confirmed by ATR-FTIR and ^13^C NMR spectra, indicating that polyacrylamide was successfully grafted on chitosan.

Figure S4c displays the X-ray diffraction of chitin and chitosan. Chitin showed two small crystalline peaks at 2θ range of 10–20°, while, in chitosan, these peaks appeared at 2θ range of 18–20° [43] and were less intense than in chitin. The grafting of chitosan with functional groups such as polyacrylamide [44] disorder this crystallinity. This behavior reveals that the obtained chitosan-grafted-polyacrylamide started to be amorphous after grafting. This change of chitosan was due to the grafting of polyacrylamide and the structure of chitosan being destroyed [44].

### 3.2. Decolorization Study of MB and MO Solutions by Chitin and CS-g-PAM

#### 3.2.1. Effect of the Initial pH on the Decolorization of the MB and MO

The pH value of the MB and MO solution are very important in the whole decolorization process, influencing not only the surface charge of chitin and the grafted chitosan, but also the availability the functional groups for dyes removal. 

**Figure 4:**
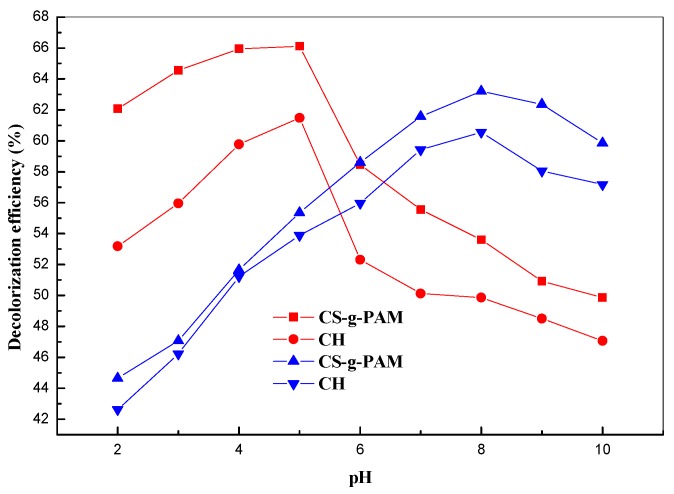
Effect of pH solution on the decolorization efficiency of MB and MO: initial MB/MO concentration: 8.0 mg/L; CS-g-PAM/CH concentration: 0.3 g·100 mL^−1^; 25 °C.

Regarding the MB decolorization efficiency, as shown in Figure 4, when initial pH value increased from 2.0 to 5.0, a slight modification in the decolorization efficiency was visible. This observation was due to the competition between the positively charged MB and H^+^ ions. In addition, a low removal was observed for the grafted chitosan and chitin. Conversely, when the initial pH value was increased from 5.0 to 10.0, the decolorization efficiency increased for MB removal (maximum at pH 8.0). This may be due to availability of the functional groups on the surface of the grafted chitosan and chitin that increased their surface complexation capability. In addition, the deprotonation of chitin and the chitosan derivative led to a good sticking between the positive charged MB and negatively charged chitin and grafted chitosan [13]. Similar results were reported for the adsorption of MB onto tea waste by Liu et al. [45] and the MB removal by 5-sulfosalicylic acid modified lignin reported by Jin et al. [46]. The authors found that the optimal pH of MB removal was 8.0.

Thus, the optimal pH for MB and MO removal was selected as pH of 8.0 and 5.0, respectively, for the present decolorization experiments. As shown in Figure 4, the maximum removal of MO was 61% by chitin and 66% by CS-g-PAM for a pH 5.0. At this pH, the amide groups of the grafted chitosan and acetamido of chitin were in protonated cationic form, and the decolorization of water-soluble MO was due to a possible attraction between the positive charge of the functional of the biopolymer beads and negative charge groups of MO. Moreover, MO can be removed via a hydrogen bonds with the OH groups of the grafted chitosan and chitin. Below pH 3.0, a decrease of decolorization process was observed because the protonation of MO led to zwiterrionic compounds, for which decolorization of MO solution was less favorable by the chitosan derivative and chitin. Above pH 5.0, decolorization efficiency drastically decreased because deprotonation of amide groups of the grafted chitosan and chitin (Figure 4). In addition, for pH above 8.0, the decolorization of MO decreased due to the presence of OH^−^ ions in basic solution as competitive ions for MO removal [47]. Similar results (pH 5) were reported by Wang et al. [48] for MO removal by the hollow chitosan microsphere adsorbent.

#### 3.2.2. Effect of Contact Time and of Initial Dye (MB and MO) Concentration 

**Figure 5:**
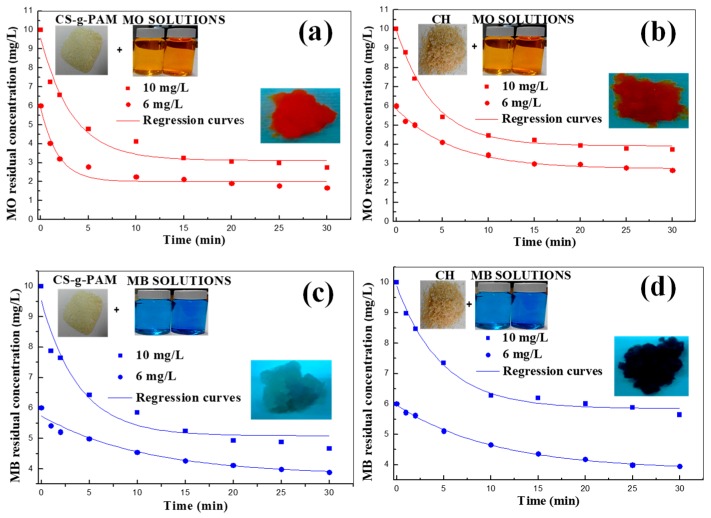
Effect of the initial dye concentration on decolorization efficiency: (**a**) MO on CS-g-PAM, (**b**) MO on CH, (**c**) MB on CS-g-PAM and (**d**) MB on CH (concentration: 0.3 g·100 mL^−1^; initial pH: 8.0 for MB and 5.0 for MO; 25 °C).

MB and MO decolorization by CH and CS-g-PAM curves versus contact time and the aspect of the two adsorbents before (at the beginning) and after decolorization (equilibrium time) are illustrated in Figure 5a–d for two selected initial dyes concentrations: 10 and 6 mg/L. As displayed in the figures, very fast decolorization was observed within the first 10 min, being then almost constant. In other words, at very low initial concentrations of the two dyes, the MB and MO decolorization by chitin and the grafted chitosan was very fast and the two dyes were adsorbed rapidly. However, during the removal of these dyes, the surface of the two powders was progressively blocked by MB and MO molecules, becoming covered very quickly. The reason is that, at the early stage, the dye molecules (MB and MO) can easily be adsorbed onto the surface of chitin and chitosan-g-PAM due to their ability and the available sites on the two adsorbents surfaces. Fan et al. [37] obtained similar results investigating the decolorization of MO by nanoscale zerovalent iron particles. The time attaining equilibrium increased with increasing concentrations. For the two dyes removal condition, the residual MB and MO concentration can be expressed by the following equation proposed by Shu et al. [49]:

(3) $$C_{dyet}=C_{ultimate}+(C_{dye0}-C_{ultimate})*\alpha *e^{-Kt},$$

$C_{dye0}$ is the initial of both dyes (MB and MO) concentration, $C_{dyet}$ is the residual of the two dyes (MB and MO) concentration at reaction time *t* (mg/L), $C_{ultimate}$ is the ultimate residual MB and MO concentration (mg/L), α is the variation coefficient for each removal of dyes and K represents the empirical rate constant (min^−1^). The different constants $C_{ultimate}$, α, and K were calculated by nonlinear regression (first order exponential decay) from the obtained experiments for MB and MO decolorization. 

Table 1 shows the obtained regression parameters such as $C_{ultimate}$, α and K as well as the coefficients of correlation $R^{2}$ of different experiments for Equation (3). The ultimate residuals ($C_{ultimate}$) of MB and MO concentrations using grafted chitosan were 5.088 mg/L and 3.125, respectively, with a selected concentration of 10 mg/L, indicating a better decolorization of MO than MB solution. The same observation was found for chitin, with $C_{ultimate}$ of 3.938 and 5.845 mg/L and k of 0.24 and 0.2 min^−1^ with a selected concentration of MB and MO of 6 mg/L, respectively. As α values were all close to the unity, Equation (3) can be transformed into Equation (4).

(4) $$C_{dyet}=C_{ultimate}+(C_{dye0}-C_{ultimate})*e^{-Kt},$$

**Figure 6:**
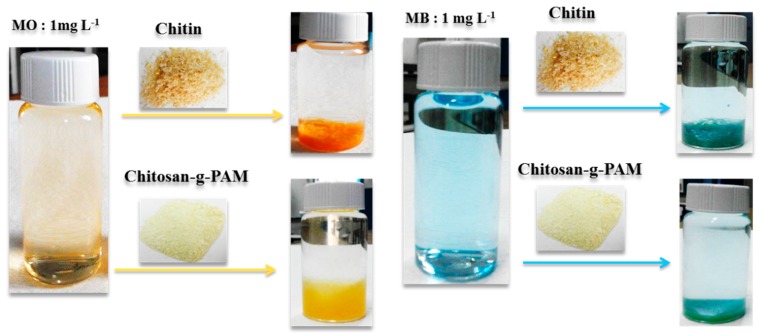
Pictures of the total decolorization of dyes solution: initial MB/MO concentration: 1.0 mg/L; CS-g-PAM/CH concentration: 0.3 g·100 mL^−1^; 25 °C.

For the removal of two dyes, their initial concentration was also decreased (Figure 6), showing MO and MB residual concentration close to zero under initial dyes concentration equal to 1 mg/L allowed for a total decolorization of two dyes solution.

High decolorization of MB and MO by eco-friendly and low-cost chitin and CS-g-PAM adsorbents was due to the presence of chelating groups on chitin and chitosan derivative structures that were responsible for this total removal of two dyes. Data regarding removal of MB and MO by other adsorbents reported in previous study are presented in Table 2 [50,51,52,53,54,55,56,57,58,59]. As shown in Table 2, various materials are used for MB and MO removal: chitin-based materials such as MnO_2_-chitin hybrid, Chitosan/Al_2_O_3_/magnetite nanoparticle composite materials and other different material composites. The adsorbents contain natural and synthetic chelating groups, which present a high level for MB and MO removal. The percent removal of these materials toward MB and MO is very high at different dose of adsorbents. Compared to those values, the CS-g-PAM and chitin showed higher removal ability for MB and MO in shorter times.

#### 3.2.3. Effect of Chitin and Chitosan-g-Polyacrylamide Concentration

Decolorization of both dye solutions was performed to fix the optimal adsorbent dose required to adsorb the MB and MO organic pollutants from the aqueous solutions at a pH 8.0 and 5.0, respectively, and contact time of 30 min for 8.0 mg/L of both dyes.

**Figure 7:**
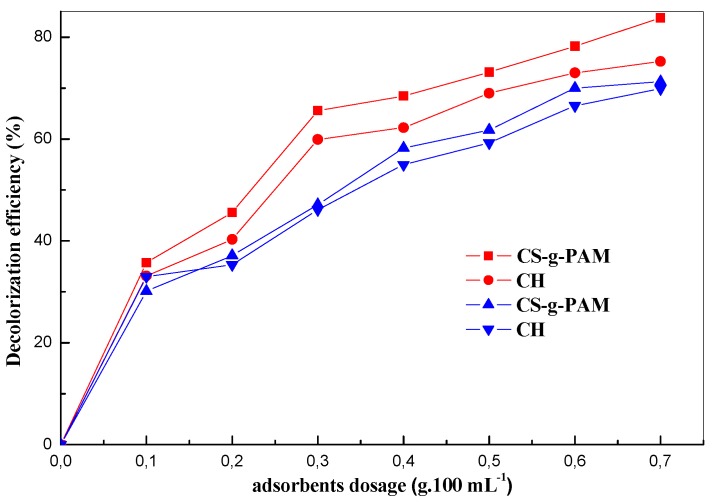
Effect of adsorbent dosage on dyes removal by CH and CS-g-PAM (pH 8.0 for MB and 5.0 for MO; initial MB/MO concentration: 8.0 mg/L; 25 °C).

The effect of the concentration of both adsorbents, CH and CS-g-PAM, on the MB and MO decolorization is shown in Figure 7. The efficiency on two dyes (MB and MO) removal for both adsorbents was found to increase with increasing amount of CH and CS-g-PAM. There was an initial sharp increase in removal efficiency due to the increasing availability of both dyes capturing sites with increased amount of CH and chitosan derivative. This suggests that the availability of the adsorption sites increased almost linearly with the increase in chitin and the grafted chitosan dose during the decolorization of both dyes. Further decolorization experiments were realized with the fixed adsorbent dosage of 0.3 g·100 mL^−1^ to better clarify the decolorization process using a low mass of adsorbents. Similar results were reported by Wong et al. on the extent of methyl orange and reactive black 5 removal by tea leaves modified with polyethyleneimine (PEI-STL) from wastewater [60]. It was found that the percentage removal increased with adsorbent dosage due to the availability of the sites of adsorption. However, for the high adsorbent dosage, the agglomeration of adsorbent leads to reducing the number of effective sites on the adsorbent surface. 

#### 3.2.4. Thermodynamic Studies

Figure S4a displays the decolorization efficiency under various temperatures. The decolorization efficiency for both dyes was higher at higher temperatures, indicating that the removal of MB and MO was relatively favorable at high temperature. The dependence of decolorization of water-soluble MB and MO on temperature can be used to calculate the value of $\Delta H^{0}$, $\Delta S^{0}$ and $\Delta G^{0}$, using the following Equations [61]:

(5) $$\Delta G^{0}=-RTLnK_{c},$$

(6) $$LnKc=\frac{\Delta S^{{}^{\circ}}}{R}-\frac{\Delta H^{{}^{\circ}}}{RT},$$

(7) $$\Delta G^{0}=\Delta H^{0}-T\Delta S^{0},$$

where $\Delta G^{0}$ is the standard Gibbs free energy changes (KJ·mol^−1^·K^−1^), T is the absolute temperature (K), *R* is the universal gas constant (KJ·mol^−1^·K^−1^), and $K_{c}$ is equilibrium constant defined as:

(8) $$Kc=\frac{Ca}{Ce}.$$

Ca is the concentration of the adsorption phase at equilibrium (mg/L) and Ce is the concentration in solution at equilibrium (mg/L).

The thermodynamic variables, summarized in Table 3, were calculated using the slope and intercept of the Van’t Hoff’s plot for the grafted chitosan and chitin for the decolorization of MB and MO solution (Figure S4a). The positive values of $\Delta H^{0}$ (+17.53 and +18.38 KJ/mol) for the decolorization of MB by chitosan derivative and chitin (+19.94 and +16.45 KJ/mol) for decolorization of MO by the grafted chitosan and chitin, respectively, confirmed the endothermic nature of the decolorization reaction, revealing that decolorization efficiency increased with an increase in the temperature. This implies that each MB and MO molecule had to displace more than one water molecule from the adsorbent surface before being adsorbed. Similar results have been reported in the literature [62,63], where the authors indicated that adsorption is based on endothermic process and favorable at high temperature, while the positive values of $\Delta S^{0}$ are indicative of increased randomness at the adsorbent–adsorbate interphase during the decolorization phenomenon, and the degree of dispersion of the process increased with increased temperature. The negative values of $\Delta G^{0}$ demonstrate the spontaneity and feasibility of MB and MO decolorization at various temperatures [64]. The increase in spontaneity with increase in temperature implies that decolorization of MB and MO by chitosan derivative and chitin was more favorable at higher temperatures. To study the thermodynamic of decolorization of water-soluble MB and MO by chitin and the grafted chitosan in detail, a modified Arrhenius-type equation related to the surface coverage ($S^{*}$), sticking probability, was used. The Arrhenius equation can be obtained using the following Equations [65]:

(9) $$S^{*}=(1-\theta )\;(e^{-\frac{E_{a}}{RT}}),$$

(10) $$\theta =\left(1-\frac{C_{e}}{C_{0}}\right),$$

(11) $$\ln(1-\theta )=\ln S^{*}+\frac{E_{a}}{RT},$$

where S* is surface coverage defined in Equations (10)–(12), $E_{a}$ is activation energy (KJ/mol), *R* is the universal gas constant (8.314 J·mol^−1^·K^−1^) and *T* is the absolute temperature (K). $C_{0}$ and $C_{e}$ are the initial and equilibrium dyes concentrations, respectively (mg/L). The parameter S* indicates the measure of the potential of an adsorbate to remain on the adsorbent indefinitely: S* > 1 indicates adsorbate unsticking to adsorbent, i.e., no adsorption; S* = 1 indicates linear sticking relationship between adsorbate and adsorbent, i.e., possible mixture of physisorption and chemisorption mechanism; S* = 0 indicates indefinite sticking of adsorbate to adsorbent, i.e., chemisorption mechanism predominant; and 0 < S* < 1 indicates favorable sticking of adsorbate to adsorbent, i.e., physisorption mechanism predominant.

The effect of temperature on the sticking probability was evaluated using a temperature range from 25 to 55 °C, by calculating the surface coverage at different temperatures (Figure S4b). The results, shown in Table 4, indicate that the probability of dye sticking to the grafted chitosan and chitin was 0 < S* < 1 for both dyes. These values confirmed the favorable sticking of MB and MO on the surface of chitosan derivative and chitin.

The activation energy ($E_{a}$) is commonly used as the basis for differentiating between physical and chemical adsorption [66]. Physical adsorption reactions are readily reversible, equilibrium is attained rapidly and thus energy requirements are small, ranging from 5.0 to 40 KJ/mol. Chemical adsorption is specific, involves stronger forces and thus requires larger activation energies (40–800 KJ/mol). Our results imply that the decolorization of MB and MO solution by the grafted chitosan and chitin required a relatively low energy ($5.0<E_{a}<40$) (Table 4), suggesting that the MB and MO were physically adsorbed onto the both materials.

#### 3.2.5. Regeneration Studies

**Figure 8:**
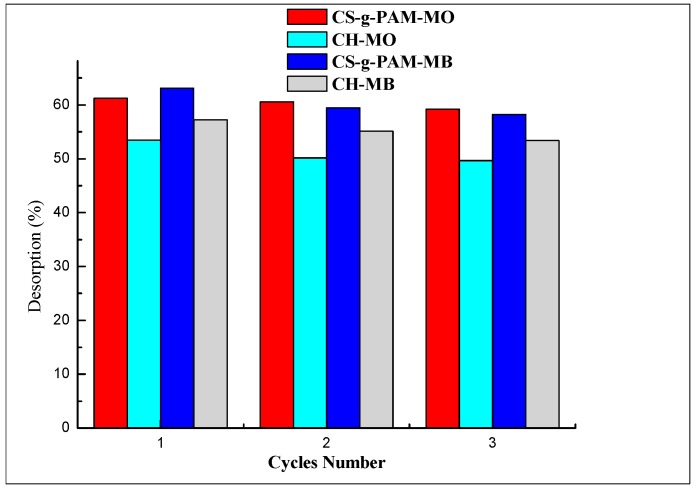
MB and MO desorption cycles (%) using 0.1 M HCl and 0.1 M NaOH as eluent, respectively. Initial MB and MO concentration: 10 mg/L.

Regeneration studies were important to investigate the potential of CS-g-PAM and CH in real applications of decolorization of MB and MO solutions. CS-g-PAM-MB and CH-MB were both recovered using 0.1 M HCl solutions; CS-g-PAM-MO and CH-MO powders were both recovered using 0.1 M NaOH solutions. The regeneration study results are illustrated in Figure 8. The desorption ratio of MB dye on the chitosan derivative and chitin in the first cycle was 55.12% and 57.24% for CS-g-PAM-MB and CH-MB, respectively, and 61.24% and 53.44% for CS-g-PAM-MO and CH-MO desorption, respectively, while, for the second and third cycles, these values decreased. This might be due to the reduction of driving force. Similar results were reported by Zeng et al. [38], whose results were similar to ours, in which the complexes obtained after MB and MO removal were successfully desorbed using HCl (0.1 M) and NaOH (0.1 M). In our study, CH and CS-g-PAM loaded MB and MO anionic/cationic dyes were desorbed using HCl (0.1 M) and NaOH (0.1 M) for 2 h. The used desorbing agents kept a high desorption percentage in the second and third adsorption–desorption cycles. Overall, due to the high recycling efficiency, CS-g-PAM and CH are both proposed for practical application.

## 4. Conclusions

This work showed the potential of chitin and chitosan-g-PAM for the decolorization of methylene blue and methyl orange soluble water solutions. Effects of critical parameters, including chitin and chitosan-g-PAM concentrations, pH, initial MB and MO concentrations, and the reaction temperature, were studied in batch experiments. The kinetic studies suggested that the decolorization process of MB and MO can be described by the first-order exponential decay kinetic model for initial concentrations of 10 and 6.0 mg/L of both dyes and can almost be completed in about 30 min. 

A higher MO removal efficiency was achieved in chitosan derivative and chitin compared to MB dye for initial concentrations of 10 and 6.0 mg/L of both dyes. A total decolorization was observed with a low initial concentration of 1.0 mg/L. The increase in reaction temperature could greatly accelerate the dye removal. The decolorization efficiency of MB and MO was strongly dependent on the pH of solution, with maximum at pH 8.0 for MB and 5.0 for MO. The CS-g-PAM and CH doses that can promote MB and MO decolorization in the adsorption system ranged from 0.1 to 0.7 g·100 mL^−1^. Further increase in chitin and chitosan-g-PAM doses caused the acquisition of methylene blue and methyl orange on the surface of the materials. The negative value of $\Delta G^{0}$ and positive value $\Delta S^{0}$ showed that decolorization of MB and MO by chitin and chitosan derivative was a spontaneous process and the positive value of $\Delta H^{0}$ indicated that decolorization of both dyes was endothermic. The activation energy $E_{a}$ less the 40 kJ/mol for the decolorization reaction of both dyes indicated the removal of MB and MO by chitin and chitosan-g-PAM was physisorption. The results show that the repeated decolorization of water soluble dye solution (three times) did not affect its desorption. 

## Figures and Tables

**Table 1:** Results of nonlinear regression of the experimental data of the decolorization of MB and MO by chitin and chitosan-g-PAM.

Material	Dye	C_0_ (mg/L)	Dose (g L^−1^)	Initial pH	T °C	$C_{ultimate}\;\mathrm{mg}/L$	K (min^−1^)	$R^{2}$	α
CS-g-PAM	MO	10	3.0	5.0	25	3.12	0.29	0.98	0.93
		6.0	3.0	5.0	25	2.01	0.55	0.98	0.97
CH	MO	10	3.0	5.0	25	3.93	0.24	0.99	0.99
		6.0	3.0	5.0	25	2.74	0.16	0.99	0.95
CS-g-PAM	MB	10	3.0	8.0	25	5.08	0.25	0.97	0.9
		6.0	3.0	8.0	25	3.80	0.09	0.98	0.88
CH	MB	10	3.0	8.0	25	5.84	0.20	0.99	0.97
		6.0	3.0	8.0	25	3.83	0.09	0.99	0.98

**Table 2:** Comparison of percent removal of MB and MO by CH and CS-g-PAM with different adsorbent materials including CH and CS-g-PAM.

Decolorizing Agent	Percent Removal (%)		[Decolorizing Agent], (g/L)	Reference
	MB	MO		
Gold nanoparticles loaded on activated carbon	>95 in 1.6 min	-	0.01	[50]
Charred Parthenium (CP)	93.4 in 3.0 h	-	0.22	[51]
Nanocomposite of Hydrolyzed Polyacrylamide Grafted Xanthan Gum and Incorporated Nanosilica	99.4 in 20 min	-	0.04	[52]
MnO_2_-chitin hybrid	99.9 in 2.5 min	-	0.35	[53]
MnO_2_-CNF hybrid	99.8 in 2.0 min	-	1.0	[54]
Tin oxide nanoparticles loaded on activated carbon (SnO_2_-NP-AC) activated carbon prepared from wood tree Pistaciaatlantica (AC-PAW)	- -	>95 in 1.0 h >95 in 1.0 h	0.015 0.08	[55]
Chitosan/Al_2_O_3_/magnetitenanoparticles composite	-	50 in 2 min	0.4	[56]
SiO_2_-Al_2_O_3_mixed-oxides	-	85 in 25 min	0.03	[57]
MnO_2_/CeO_2_catalyst	-	90 in 10 min	1.0	[58]
NiFe layered double hydroxides (LDHs)	-	92 in 10 min	0.02	[59]
Chitin/CS-g-PAM		≈100/100 in 30 min	3.0	This study

**Table 3:** Thermodynamic parameters for the decolorization of MB and MO by chitin and chitosan-g-PAM (pH 8.0 for MB and 5.0 for MO; initial MB/MO concentration: 4.0 mg/L; mass of CH and CS-g-PAM: 0.3 g·100 mL^−1^).

Material	Dye	$\Delta H^{0}\;(\mathrm{KJ}/\mathrm{mol})$	$\Delta S^{0}\;(\mathrm{KJ}\cdot {\mathrm{mol}}^{-1}\cdot K^{-1})$	$\Delta G^{0}\;(\mathrm{KJ}/\mathrm{mol})$	T (°C)
CS-g-PAM	MO	+19.94	+0.07	−2.11	25
				−2.85	35
				−3.59	45
				−4.33	55
CH	MO	+16.45	+0.06	−1.17	25
				−2.33	35
				−2.84	45
				−3.55	55
CS-g-PAM	MB	+17.53	+0.06	−1.24	25
				−1.87	35
				−2.50	45
				−3.13	55
CH	MB	+18.38	+0.06	−0.69	25
				−1.33	35
				−1.97	45
				−2.61	55

**Table 4:** The values of S* and Ea for the decolorization of MB and MO by chitin and chitosan-g-PAM (pH 8.0 for MB and 5.0 for MO; initial MB/MO concentration: 4.0 mg/L; mass of CH and CS-g-PAM: 0.3 g·100 mL^−1^).

Material	Dye	$S^{*}$	$E_{a}\;\left(KJmol^{-1}\right)$	$R^{2}$
CS-g-PAM	MO	0.00048	15.80	0.96
CH	MO	0.0020	12.34	0.99
CS-g-PAM	MB	0.0023	12.55	0.97
CH	MB	0.0026	12.48	0.96

## Supplementary Materials

The following are available online at http://www.mdpi.com/1996-1944/12/3/361/s1, Figure S1. ^13^C NMR spectra of chitosan (a) and chitosan-g-polyacrylamide (CS-g-PAM) (b), Figure S2. TGA (a) and DTG (b) spectra of chitosan and chitosan-g-polyacrylamide (CS-g-PAM). Figure S3. DSC spectra of chitosan and chitosan-g-polyacrylamide (CS-g-PAM). Figure S4. SEM micrographs of chitin (a) and chitosan (b); XRD of chitin and chitosan (c). Figure S5. Arrhenius plot of $LnK_{c}$ versus $\frac{1}{T}$ for the decolorization of MB and MO by CS-g-PAM and CH: initial MB and MO concentration: 5.0 mg/mL; CS-g-PAM and CH concentration: 0.3 g·10 mL^−1^ (b); plot of ln(1−θ) versus $\frac{1}{T}$ for the decolorization of dyes solution: initial MB, MO concentration: 10 mg/L; CS-g-PAM and CH concentration: 0.3 g·10 mL^−1^ at 25 °C (b).

## Abbreviations

CS	Chitosan
CH	Chitin
AM	Acrylamide
CS-g-PAM	Chitosan-grafted-polyacrylamide
MB	Methylene blue
MO	Methyl orange
%G	Percentage of grafting
^13^C NMR	^13^Carbon nuclear magnetic resonance
FTIR	Fourier transform infrared
TGA	Thermogravimetric analysis
DTGA	Differential Thermogravimetric analysis
DSC	Differential Scanning Calorimetry
DE	Decolorization efficiency (mg/L)
$C_{dyet}$	The residual MO and MB concentration at reaction time t (mg/L)
$C_{\mathrm{dye}0}$	The initial MO and MB concentration (mg/L)
$C_{\mathrm{ultimate}}$	The ultimate residual MO and MB concentration (mg/L)
α	The variation coefficient
K	The empirical rate constant (min^−1^)
T	Temperature (°C)
Ca	The adsorbent phase concentration at equilibrium (mg/L)
Kc	The equilibrium constant
$\Delta G^{0}$	Standard free energy (KJ/mol)
$\Delta H^{0}$	Standard enthalpy change (KJ/mol)
$\Delta S^{0}$	Standard entropy change (KJ/mol∙K)
$S^{*}$	The sticking probability
$E_{a}$	The activation energy (KJ/mol)
