# Supplementary material for: Functional Chitosan Derivative and Chitin as Decolorization Materials for Methylene Blue and Methyl Orange from Aqueous Solution
Source: Materials (Basel). 2019 Jan 24;12(3):361. doi: 10.3390/ma12030361 (PMC6384594; doi:10.3390/ma12030361)
Supplement: Supplementary file 1 [file materials-12-00361-s001.pdf]

materials

MDPI

Article

Functional Chitosan Derivative and Chitin as Decolorization Materials for Methylene Blue and Methyl Orange from Aqueous Solution

Abdelkader Labidi [1,2], Asier M. Salaberria [3], Susana C. M. Fernandes [4], Jalel Labidi [3,*] and Manef Abderrabba [1]

1 Preparatory Institute of Scientific and Technical Studies of Tunis, University of Carthage, Sidi Bou Said road, B.P. 51 2070, La Marsa, Tunisia; abdelkaderlabidi0907@gmail.com(A.L.); abderrabbamanef@gmail.com(M.A.)
2 Chemistry Department, University of Sciences of Tunis, El Manar University, B.P: 248, El Manar II, 2092, Tunis, Tunisia.
3 Department of Chemical and Environmental Engineering, University of the Basque Country (UPV/EHU), Plza. Europa1, 20018 Donostia-San Sebastian, Spain; asier.martinez@ehu.eus
4 CNRS/ Univ Pau & Pays Adour, Institut des Sciences Analytiques et de Physico-Chimie pour l'Environnement et les Materiaux, Umr 5254, 64000, Pau, France; susana.fernandes@univ-pau.fr
* Correspondence: jalel.labidi@ehu.es

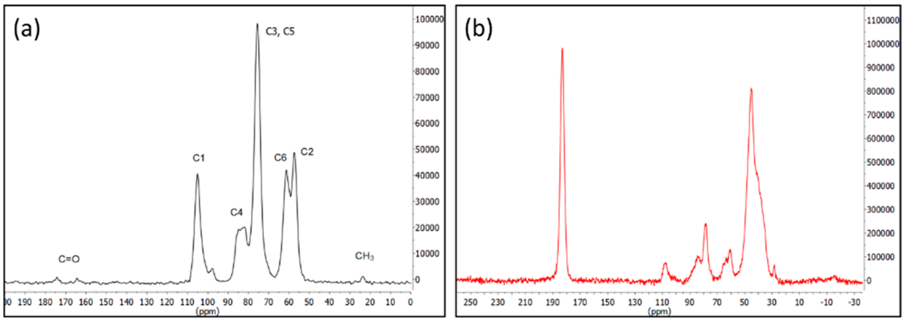

Figure S1. ^{13}C NMR spectra of chitosan (**a**) and chitosan-g-polyacrylamide (CS-g-PAM) (**b**).

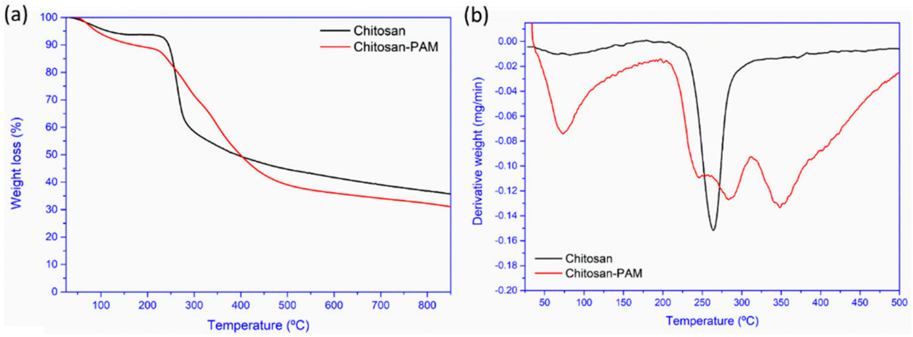

Figure S2. TGA (**a**) and DTG (**b**) spectra of chitosan and chitosan-g-polyacrylamide (CS-g-PAM).

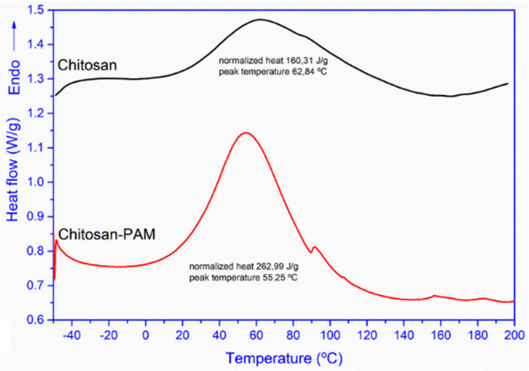

Figure S3. DSC spectra of chitosan and chitosan-g-polyacrylamide (CS-g-PAM).

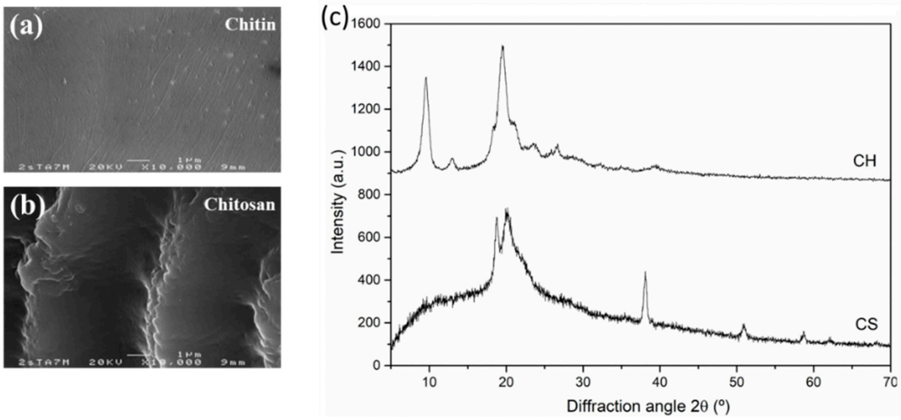

Figure S4. SEM micrographs of chitin (**a**) and chitosan (**b**); XRD of chitin and chitosan (**c**).

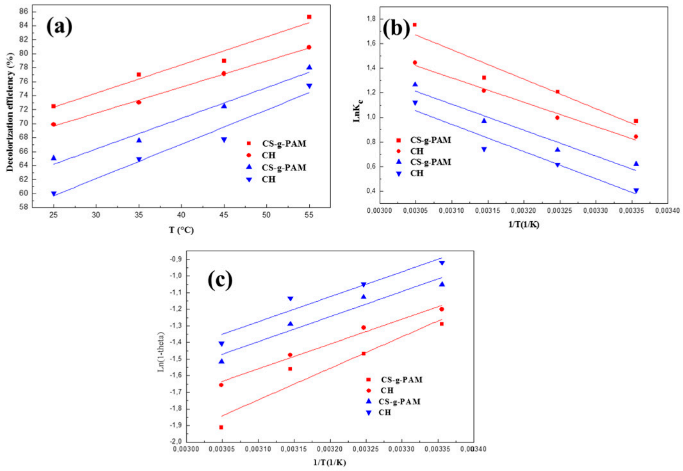

Figure S5. Arrhenius plot of LnK_c versus $\frac{1}{T}$ for the decolorization of MB and MO by CS-g-PAM and CH: initial MB and MO concentration: 5.0 mg/mL; CS-g-PAM and CH concentration: 0.3 g·10mL^{-1} (**a**); plot of $\ln(1-\theta)$ versus $\frac{1}{T}$ for the decolorization of dyes solution: initial MB, MO concentration: 10 mg/L; CS-g-PAM and CH concentration: 0.3 g·10mL^{-1} at 25 °C (**b**).
